# Geometric basis of action potential of skeletal muscle cells and neurons

**DOI:** 10.1515/biol-2022-0488

**Published:** 2022-09-16

**Authors:** Qing Li

**Affiliations:** Department of Function, ShiJiaZhuang Traditional Chinese Medical Hospital, No. 233, ZhongShan West Road, ShiJiaZhuang, HeBei Province 050051, China

**Keywords:** action potential, skeletal muscle cells, synaptic connection of neurons, space curved manifolds, time curved manifolds

## Abstract

Although we know something about single-cell neuromuscular junctions, it is still unclear how multiple skeletal muscle cells coordinate to complete intricate spatial curve movement. Here, we hypothesize that skeletal muscle cell populations with action potentials are aligned according to curved manifolds in space (a curved shape in space). When a specific motor nerve impulse is transmitted, the skeletal muscle also moves according to the corresponding shape (manifolds). The action potential of motor nerve fibers has the characteristics of a time curve manifold, and this time-manifold curve of motor nerve fibers comes from the visual cortex in which spatial geometric manifolds are formed within the synaptic connection of neurons. This spatial geometric manifold of the synaptic connection of neurons originates from spatial geometric manifolds outside nature that are transmitted to the brain through the cone cells and ganglion cells of the retina. The essence of life is that life is an object that can move autonomously, and the essence of life’s autonomous movement is the movement of proteins. Theoretically, because of the infinite diversity of geometric manifold shapes in nature, the arrangement and combination of 20 amino acids should have infinite diversity, and the geometric manifold formed by the protein three-dimensional spatial structure should also have infinite diversity.

## 1 Introduction

In 1971, the place cells (hippocampal complex spike cells) were discovered by recording the activity of the hippocampus by the microelectrodes fixed to the skull of the rats [1]. When the rat ran to a specific area, these cells always showed activation, while other cells activated when it ran to another area. In 2005, the grid cells were discovered in the dorsocaudal medial entorhinal cortex (dMEC) by recording the spike activity of the fired cell in this area of rats, which is activated whenever the animal’s position coincides with any vertex of a regular grid of equilateral triangles spanning the surface of the environment [2]. Some cells respond to specific location areas of the environment, like place cells in the hippocampus. Furthermore, in 2015, the study indicated that the environmental boundaries influence grid cell firing, where the grid orientation, scale, symmetry, and homogeneity are affected by environmental geometry [3]. The hexagonal grid symmetry is permanently broken in highly polarized environments such as squares and trapezoids, the pattern being more elliptical and less homogeneous. In recent years, intrinsic, recurrently connected continuous attractor networks (CANs) of neurons have been put forward as a possible substrate of the grid pattern [4]. In 2022, a study with simultaneous recordings from many hundreds of grid cells by taking advantage of recently developed high-site-count neuropixels silicon probes that allow spike activity to be recorded simultaneously from thousands of cells in freely moving rats, and subsequent topological data analysis, show that the joint activity of grid cells from an individual module resides on a toroidal manifold, as expected in a two-dimensional CAN [5]. Positions on the torus correspond to the moving animal’s position in the environment. Individual cells are preferentially active at singular positions on the torus. This provides the first evidence for mankind where the network dynamics of neurons (visualization of CAN dynamics) on a toroidal manifold illustrates the population-level activity of grid cells. In a natural evolution, the cortical cortex of the brain has a division of labor; therefore, the cerebral cortex of higher animals is divided into many areas, such as the motor center controlling skeletal muscle movement and the visual center cortex controlling vision. Although the evidence of the manifold of neurons only appears in the hippocampus and dMEC, other nerve centers have common characteristics, and the visual center and motor center are no exception.

The evidence for the joint spike activity of grid cells from an individual module (a two-dimensional CAN) residing on a toroidal manifold raises questions about how this grid representation (or visual center neurons and motor center neurons representation) might be generated and maintained. Here, we propose that this toroidal manifold of CAN is generated by the spatial geometric manifold of the synaptic connection of neurons which originates from spatial geometric manifolds in outside nature that are transmitted to the brain through the cone cells and ganglion cells of the retina. Furthermore, the toroidal manifold of CAN is only one of all types of curve manifolds. Other types of manifolds will be confirmed in the future, so a curve manifold has universal significance.

The baseline for the hypothesis is: what is the essence of life, that is, what is the fundamental difference between living things and non-living things? There are several answers to this question. For example, the essence of life is the genetic code DNA [6], protein, or cell that can carry out the material exchange (metabolism) or the negative entropy that can be studied by physical and chemical methods [7]. All of these characteristics of life do not exist in non-biological bodies. All the above are true, but there are many answers. In any case, there was only one answer to this question. In our opinion, the essence of life is that it is an object that can move autonomously. In any case, all the contents of this article are hypotheses that have not been confirmed by experiments, but they can give us some positive hints.

So, what is autonomous movement? For example, the motion state of an inanimate body (non-organism), such as a basic particle, depends on its interaction with other substances; therefore, its motion is random, that is, quantized motion, so its motion is not autonomous motion. Here, we define autonomous motion as independent of other substances and can actively and consistently perform any space curve shape motion. We were unable to find a non-organism that could move autonomously in nature. A cell is an object that can perform autonomous movement; therefore, the essence of life is a cell. The autonomous movement of cells can also be divided into simple and complex movements, and organisms have evolved from single to multiple cells. A single cell performs simple autonomic movements, and multiple cells perform complex and fine autonomic movements. How do cells complete their autonomous movements? This was the main focus of this study.

## 2 Results

### 2.1 Why do cells move autonomously while other objects cannot?

The evolution of nature from inorganic to organic matter is complex. The origin of the cells is the main sign of this evolution. Let us use a single-cell organism to explain why the cell is the basic working unit of autonomous movement. For example, although bacteria are single-celled organisms, their complex structures are used to maintain their autonomous movement.

The cell membrane has the function of selective permeation and completes material exchange inside and outside the cell together with the cell wall [8,9]. Respiratory enzymes on the membrane participate in the energy metabolism of cells [10,11]. The cytoplasm is the primary site of bacterial metabolism [12,13]. The ribosome is a site of protein synthesis [14,15]. The Golgi matrix forms the basis for protein processing, sorting, and transportation [16,17]. The nucleoplasm is composed of genetic material DNA, which controls the life activity of bacteria and is the material basis of its genetic variation [18,19]. Flagellae are filaments attached to the surface of bacteria and are the motor organs of cells [20,21].

All the structures of the cell serve the autonomous movement of the cell. The ion channel of the cell membrane controls the cell to perform autonomous movement [22,23]. It transmits external information to the cell through the amount of ion flow, and the cell completes the corresponding movement according to the amount of information flow of the amplitude motion.

The flagellum motor contains a proton pump, which can transfer hydrogen ions to drive the rotation of the proton pump [24,25], convert chemical energy into mechanical energy, transmit torque to the flagellum joint device, and then drive the flagellum filaments to drive bacteria like a propeller [26]. Therefore, the autonomous movement of bacterial cells is simple, and their movement state and direction depend on the surrounding ionic environment. Proteins are the material basis for cellular work and a substance that exercises autonomic motion. Why do proteins exist on this material basis? The reasons for this are as follows: (1) Its physical and chemical properties, such as stretch, flexibility, and elasticity, enable it to complete a variety of complex and delicate movements [27,28]. (2) A protein are polymers composed of 20 different amino acids [29]. Its amino acid sequence and spatial position are almost endless [30], which indicates that it can complete the movement of infinite geometry and perform various physiological functions in cells.

### 2.2 Multicellular autonomous movement

#### 2.2.1 Characteristics and functions of multicellular ion structure

The evolution of life from single cells to multiple cells is to complete more complex and fine autonomous movements. As mentioned earlier, the ion flow drives cells to complete autonomous movement, and the amount of ion flow determines the range of cell movement. Here, we take metazoan animals as an example because they have the ion channel structure of a sodium–potassium pump. Furthermore, we take human ion channels as an example because people can perform the most complex and fine autonomous movements. The brain dominates the human skeletal muscle movement. The structure and function of ion channels that complete the excitation–transfer coupling of nerve skeletal muscles have been systematically and completely studied [31]. We used a single cell as an example to illustrate this transfer coupling process.

In the axoplasm of axon terminals of motor nerve fibers, there are a large number of vesicles with a diameter of approximately 50 nm. ACh is contained in the vesicles, and ACh stored in each vesicle is usually constant [32,33]. When ACh is released by exocytosis in vesicles, it is called a quantum release. When there is a nerve impulse at the nerve endings, under the action of local membrane depolarization caused by an action potential, a large number of vesicles approach the axon membrane, and all ACh in the vesicles enters the junction space through the fusion of the vesicle membrane and axon membrane [34,35]. It is estimated that approximately 200–300 vesicle contents can be discharged by the arrival of action potential [36]. When ACh reaches the endplate membrane surface of skeletal muscle cells through the junction gap, it immediately interacts with the channel protein (sodium–potassium pump) on the membrane α-subunit binding, causing conformational changes in protein molecules, resulting in channel opening, sodium ion influx, and potassium ion outflow, resulting in endplate membrane depolarization [37,38]. This junction transmission maintains a one-to-one relationship; every time a nerve impulse reaches the end of the motor fiber, it reliably excites the muscle cells and produces a contraction.

Skeletal muscle is composed of a large number of muscle fibers arranged in parallel. Each muscle fiber is a muscle cell, and thousands of myofibrils exist in each muscle cell. Myofibrils take the sarcomere as the unit, and the finer structure of the sarcomere is a myofilament. Myofilaments are composed of thick myofilaments (main component of myosin) [39] and fine myofilaments (main component actin) [40,41]. The action potential generated by depolarizing the endplate membrane of muscle cells increases the calcium ion concentration in muscle plasma. Troponin binds a sufficient number of calcium ions to cause a conformational change in the troponin molecule, which is then transmitted to myosin. The conformation of the latter also changes and its structure has a certain torsion, which leads to the binding of actin and transverse bridge. The two bind, twist, and dissociate and then combine, twist, and dissociate to trigger the cross-bridge cycle [42,43]. This cycle causes fine muscle filaments to slide between thick muscle filaments to complete muscle contraction. The transverse bridge functions as an ATPase that can decompose ATP and provide energy.

#### 2.2.2 Mechanism of multicellular autonomic movement

(1) A basic question arises. How can skeletal muscle complete complex and fine autonomous motion, that is, curve or straight-line motion with an arbitrary shape in space? However, this remains unclear. As previously mentioned, action potentials drive cell movement. For a single cell, we clearly understand the process of completing autonomous movement through ion exchange. How do the action potentials of multiple cells coordinate and complete the various forms of autonomous movement? Theoretically, when a nerve impulse in the cerebral cortex is transmitted, each cell is given a different motion amplitude and direction through different action potentials. Therefore, thousands of cells with different motion amplitudes and directions complete a fine movement of skeletal muscle, that is, a specific curve-shaped movement. Such action potentials should have the following characteristics. (1) They are not all or none but increase with an increase in stimulation [44]. (2) The resulting cell movements had different directions. (3) They cannot perform long-distance transmissions on the cell membrane.

**Figure 1:**
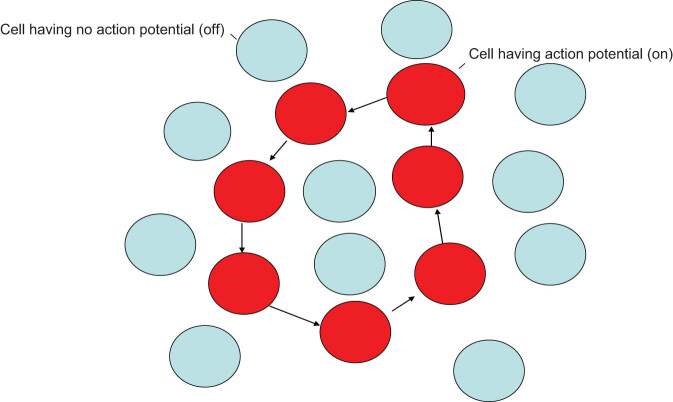
The cell populations with action potentials are alligned according to a curved manifolds on space (a curved shape on space) and the skeletal muscle also moves according to this corresponding shape (manifolds) when an specific nerve impulses are transmitted. Other cells outside this manifolds are in a state without action potential. The cells in red are cells that have an action potential and forms a special geometric manifold. The cells in blue are cells that have no action potential and not manifolds formed.

However, the complexity of this mechanism is unimaginable. The subtlety of nature involves describing complex things with simplicity. Experimental studies have confirmed that skeletal muscle action potentials follow the principle of simplicity. The skeletal muscle action potential has the characteristics of all or none; that is, the action potential of each cell does not change with the stimulation intensity and conduction distance. Therefore, when nerve impulses are transmitted, each cell can only have two possible states: either producing action potentials or not producing action potentials. How do multicells perform complex movements based on these two states? Here, I propose a hypothesis that cell populations with action potentials are aligned according to curved manifolds in space(a curved shape in space) and that the skeletal muscle also moves according to this corresponding shape(manifolds) when a specific nerve impulse is transmitted. Cells outside this manifold are in a state without an action potential. As shown in Figure 1, different nerve impulses produce different curve manifolds, that is, the switching states of the action potentials of different cell groups. Here, all skeletal muscle cells can complete various complex and fine movements as long as each cell executes these two on-or-off switching states. It can be seen that the direction of motion of a single cell is determined by these manifolds. We can also refer to this manifold of multicellular action potentials as the spatial geometric distribution or spatial summation distribution of the action potentials.

**Figure 2:**
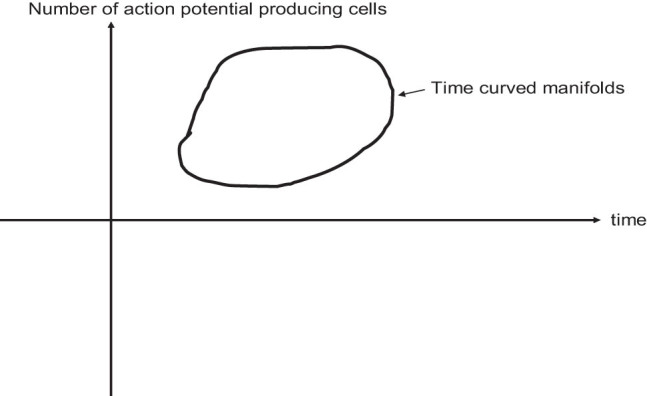
*X*-axis is time, *Y*-axis is the number of cells generating action potential, time-manifold curve is a function of independent variable *x* with respect to dependent variable *y*, and this time-manifold curve corresponds to space manifold curve.

(2) Therefore, the following question arises. Although the excitation transmission between the nerve endings of motor nerve fibers and muscle cells is 1-to-1, the relationship between the trunk of motor nerve fibers and muscle cells is not 1-to-1. How do nerve impulses in the cerebral cortex pass through the trunk of nerve fibers? Here, the concept of temporal geometric distribution (time-curved manifolds) is proposed, in view of the previous concept of spatial geometric distribution. In other words, the action potential of motor nerve fibers has the characteristics of a time-curved manifold, so how can we understand this time manifold? Because the action potential propagation speed of the nerve fibers is constant, the size of this time manifold is defined by the number of excited muscle cells per unit time, as shown in Figure 2. This time-manifold curve of motor nerve fibers corresponds to the space-manifold curve of the action potentials of skeletal muscle cells.

(3) Theoretically, the movement of skeletal muscle is based on the manifold shape formed by the action potential of the skeletal muscle cell group triggered by nerve impulses of motor nerve fibers. This manifold is formed by the spatial distribution of cells with an action potential (on), while the other cells do not produce an action potential (off). Each manifold is specific, and the actual movement of the skeletal muscle corresponds to this manifold. However, if the skeletal muscle cell group is closely connected in space, and if this manifold is formed by a group of cell groups in space, the movement of the skeletal muscle cannot be realized.

The same is true. The skeletal muscle consists of a large number of bundles of muscle fibers arranged in parallel. Each muscle cell is a muscle fiber, and each muscle fiber is slender and cylindrical. This structural feature determines that the movement of skeletal muscle has only two linear movements, contraction (shortening) and elongation, but cannot complete the complex and fine manifold movement. Multiple skeletal muscle cell groups are required to participate to complete this manifold movement. With the cooperation of the bone and joints, these cell groups coordinate to complete this manifold movement.

Here, we propose the following hypothesis: Each group of cells can complete only a simple linear motion of shortening and lengthening. The amplitude of this linear movement in each group of cells was determined by the number of cells stimulated to produce the action potential. The greater the number of cells, the greater the degree of contraction and relaxation (lengthening and shortening) of muscle cells.

To complete the three-dimensional manifold motion, three groups of cells are needed. One group of cells controls up and down movements, the second group controls left and right movements, and the third group controls front and rear movements. The three groups of muscle cells are different cell groups that cannot replace but have cross-action each other. Because each group can only move in a straight line of front and rear direction, the three groups require the participation of bones and joints to complete the three-dimensional manifold movement: for example, in the three-dimensional movement of middle fingertip cell groups, the group of cells of flexor digitorum profundus and flexor digitorum superficialis controlling the front and rear directions movements [45], the group of cells of palmar interossei and dorsal interossei controlling the left and right directions movements [46], and the group of cells of lumbricales controlling up and down directions movements [47]. The movements of three cell groups require joint participation. Their contraction and relaxation take the joints as the fulcrum to pull and control each cell group of the front and rear movement to make them perform front, rear, left, right, up, and down movements. When different degrees of motor nerve fiber impulses come, the different number of cells excited to produce action potential in each of the three groups of cell groups determines the degree of fingertip up and down, front and back, and left and right movements, resulting in various complex geometric manifold movements. This motion of space manifold curve of action potentials of skeletal muscle cells also corresponds to the time-manifold curve of motor nerve fibers.

(4) As mentioned earlier, the three-dimensional manifold movement of skeletal muscle moves according to the manifold shape formed by the action potential of the skeletal muscle cell group triggered by nerve impulses of motor nerve fibers, and motor nerve fibers carry this geometric manifold information in the form of time manifold, so there is no doubt that this manifold information comes from the motor center cortex of the brain, so how does the motor cortex transmit this information? Here, we propose the following hypothesis: as mentioned earlier, the essence of life is autonomous motion, and the essence of autonomous motion is geometric manifold motion. Therefore, a geometric manifold is the most basic determinant of life. Therefore, the manifold information of the cerebral cortex comes from nature and acts on our sensory system.

The external geometric manifold information is transmitted to the visual cortex through photons.

When an external object with a geometric shape is projected onto the eye through the optical system, the photon binds to the photoreceptor (rhodopsin) [48] on the membrane of the outermost photoreceptor cell (mainly cone cell) of the retina. The conformation of the latter changes activates the opening of the chemically gated Na+ channel of the cell membrane and generates a hyperpolarized receptor potential [49]. This receptor potential spreads to its axon terminals with electrical tension, affects the release of transmitters, and triggers the formation of an action potential in its lower ganglion cells, which has the cumulative characteristics of quantity and does not have the characteristics of all or no action potential. In fact, cones and bipolar cells do not have the ability to generate action potentials, but only ganglion cells. There are approximately six million cone cells and 1.2 million ganglion cells in the human retina [50]. Each ganglion cell is connected to 40,000–60,000 cone cells through dendrites. Ganglion cells form optic nerve fibers through long axons and project to the visual cortex [51].

When objects with different geometries project photons to the visual system, the number of photons absorbed by the cones is different, and the number of ganglion cells producing action potential is also different.

Just as the motor nerve fibers mentioned above have the characteristics of a time manifold, the cone cells and ganglion cells also have the characteristics of a time manifold when transmitting external space manifold information, where spatial geometric distribution in outside nature corresponds to temporal geometric distribution (time curved manifolds) of the cone cells and ganglion cells. The time manifold of cone cells is calculated by the number of photons bound by visual receptors per unit of time, and the time manifold of ganglion cells is calculated by the number of cells producing action units per unit of time. Thus, the geometric manifold of an external space can be described by the time manifold of cone and ganglion cells. This time manifold can be projected by the ganglion cells to the visual cortex through the optic nerve.

(5) How does the time-manifold information of ganglion cell action potentials transfer to the visual central cortex? Unlike the action potential geometric manifold of skeletal muscle cells, which have wide spatial ductility, the neurons in the visual cortex are closely connected. The limitation of the skull makes it impossible for these neurons to have motion space. Therefore, the time manifold of ganglion cells is stored in the visual cortex in a different way. Here, we propose the hypothesis that the synaptic connection of neurons of the visual cortex will form a spatial geometric manifold, which corresponds to the temporal geometric manifold of the ganglion action potential.

When ganglion cells (optic nerve fibers) transmit nerve impulses to the visual cortex through the lateral geniculate body, which has multiple synaptic connections with the visual cortex, a geometric manifold of synaptic connections between neurons in the visual cortex is formed. Owing to the different manifold shapes in the external space, the geometric manifold shapes of synaptic connections between neurons are also correspondingly different, and the two have a one-to-one relationship. How does the geometric manifold of synaptic connections form?

**Figure 3:**
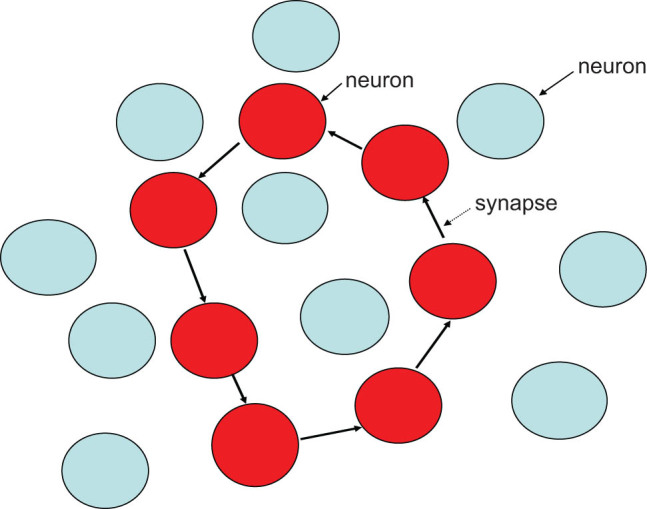
When the first neuron generating the action potential conducts excitation to the neurons adjacent to it, its conduction direction is also specific, which is transmitted according to the direction specified by this special geometric manifold, That is, it is only transmitted to the adjacent cells in the direction consistent with the manifold shape, not to the adjacent cells in other directions. When this transmission is continuous, a synaptic connection between neurons with a special geometric manifold is formed. The neurons in red are neurons that have an action potential and form synapses in a particular direction. The neurons in blue are neurons that have no action potential and do not form synapses in a particular direction.

When the impulse of an optic nerve fiber with a special time-geometric manifold is transmitted, the action potential generated by the visual cortex neurons also has a time sequence. When the first neuron generating the action potential conducts excitation to the neurons adjacent to it, its conduction direction is also specific, which is transmitted according to the direction specified by this special geometric manifold; that is, it is only transmitted to the adjacent cells in a direction consistent with the manifold shape, and not to the adjacent cells in other directions. When this transmission is continuous, a synaptic connection between the neurons with a special geometric manifold is formed (Figure 3).

When a new special geometric manifold from the outside is transmitted through the impulse of optic nerve fibers, there is no existing synaptic connection with this shape between neurons; therefore, it is necessary to form a new synaptic connection with this special shape. It is known that the synaptic connections between neurons are mainly axon–cell body connections, which are composed of presynaptic membranes, synaptic spaces, and postsynaptic membranes. The action potential transmission of this synapse is unidirectional and mediated by neurotransmitters.

The synaptic structure is mainly composed of proteins. Therefore, the formation of a new synaptic geometric manifold loop requires the synthesis of new proteins, which is a complex process. Studies have shown that the formation of an axon-somatic synapse is mainly mediated by the axon guidance pathway. This axonal guidance mechanisms are mediated by many molecules, such as netrin (midline axonal guidance), Neuroglian and Fasciclins (longitudinal axonal guidance) [52], and so on.

The formation of a new synaptic geometric manifold loop cannot be generated by only one impulse transmission; it can only be generated by many times of the same stimulation. Therefore, the synaptic geometric manifold does not have infinity; that is, the manifold shape generated and consolidated between neuronal synapses completely depends on the stimulation intensity and duration of this shape that emerges in the visual pathway. In contrast, if a new synaptic manifold cannot be consolidated and strengthened for a long time, this manifold may be gradually lost, which is called memory loss.

Long-term and short-term memory can be explained using this concept [53]. Long-term memory is the generation of a synaptic manifold that represents the synthesis of new proteins. Short-term memory implies that there is no synthesis of new proteins. It is a new external manifold stimulation, which is similar to or consistent with the existing brain synaptic manifold. On the contrary, if a new synaptic manifold cannot be consolidated and strengthened for a long time, then this manifold may be gradually lost, which is called memory loss.

From this, we can see that the synaptic manifold of neurons is the basis and essence of memory. The more synaptic manifolds, the stronger the memory and logical thinking ability.

In natural evolution, the cortical cortex of the brain has a division of labor; therefore, the cerebral cortex of higher animals is divided into many areas [54], such as the motor center controlling skeletal muscle movement and the visual center cortex controlling vision. How do they relate, or how does the movement center cortex start the geometric manifold movement of the skeletal muscle? It is speculated that they have an intermediate nerve nucleus connection similar to nerve fibers or multiple synaptic connections with each other. This needs further research. As mentioned earlier, how the action potential manifold conducted by visual nerve fibers leads to the new synthesis of neuronal proteins and synapse formation is still a mystery now.

## 3 Protein is the basis of autonomous movement of life

As previously mentioned, the essence of life’s autonomous movement is the movement of proteins. Only 20 basic amino acids make up proteins, but their arrangement and combination have the possibility of infinite diversity, which also conforms to the law of nature to express complex diversity with simplicity. The geometric manifold movement of skeletal muscle is mainly performed by myosin and actin on muscle fibers, with the participation of other proteins [55,56]. With the aid of bones and joints, only a limited number of proteins can complete various complex manifold movements (but cannot move on an infinite number of manifold shapes). However, theoretically, owing to the infinite diversity of geometric manifold shapes in nature, the arrangement and combination of 20 amino acids should have infinite diversity, and the geometric manifold formed by the protein three-dimensional spatial structure should also have infinite diversity. The transformation from one manifold to another (change in the autonomic movement state of life) only requires a change in the arrangement and combination of amino acids.

Because DNA is a genetic material and protein synthesis uses it as a template [57], the infinite diversity of arrangement and combination of 20 amino acids also determines the infinite diversity of arrangement and combination of four nucleotides (infinite diversity of genes), which also follows the natural law of expressing complexity with simplicity.

Theoretically, if a large number of proteins and genes are placed in a cell or arranged in a system from gene to protein, the movement of proteins from one shape to another will become extremely complex in this organism. Living organisms use cells as the working unit to solve this problem. There are fewer proteins and genes in each cell, and the division of labor of each cell is the same or different. Instead of the direct change of a group of three-dimensional manifolds composed of many proteins, in the connection between cells, the change in manifolds can be realized only by increasing and decreasing the number of cells. Therefore, physiological replication and apoptosis of cells are essential to meet the needs of protein manifold changes. Manifold changes in cells have the following advantages. (1) Each cell has fewer genomes and produces fewer proteins, and its time and process are simplified. (2) For manifold changes containing more proteins, only the replication and apoptosis of their cells can be changed without direct change. Here, the protein–protein interaction (a small group of proteins connected with a small group) replaces the overall connection of a large group of proteins.

From these reasons, it can be seen that the essence of life is autonomous movement, and the material basis of autonomous movement is protein. Therefore, autonomous movement is a change in the geometric manifold of a protein. Furthermore, the protein geometric manifold is the essence and basis of all life phenomena. Gene expression, gene regulation (gene–protein interaction), and protein synthesis regulation are aimed at changing this geometric manifold.

Under the stimulation of various external pressures, when skeletal muscle or other organs need to move in a new geometric manifold that does not exist in the synaptic connection of their own nerve center, the three-dimensional manifold structure of the protein needs to meet and merge into this manifold shape, so the gene mutations that control protein synthesis occur; it has been suggested that transposons can change genome size and cause mutations, which may cause changes in the geometric manifold of proteins. When mutations or other factors lead to the dissociation of protein–protein interactions, multiple cells die, resulting in a large area of protein fracture (collapse of geometric manifold), and the organism begins to senescence or death.

## 4 Perspectives

In the future, to confirm the properties of the spatial geometric manifolds of the synaptic connection of neurons of the visual cortex, probes are needed where individual spikes can be reliably distinguished in temporal resolution and the action potentials of large-scale neuronal populations in the local field of the visual cortex can be measured [58]. Furthermore, when hundreds and thousands of well-isolated single neurons are simultaneously recorded from different fields of the visual cortex in awake animals, surprising experimental results may be obtained. By then, it becomes possible that a direct observation on the spatial summation distribution of action potentials with all or none pattern within the group of skeletal muscle cells.
